# Supplementary material for: Genome-Wide Identification of WRKY Gene Family and Functional Characterization of CcWRKY25 in Capsicum chinense
Source: Int J Mol Sci. 2023 Jul 13;24(14):11389. doi: 10.3390/ijms241411389 (PMC10379288; doi:10.3390/ijms241411389)
Supplement: Supplementary file 1 [file ijms-24-11389-s001.zip › Figure S1The detection of T2 generation transgenic Arabidopsis.pdf]

Figure S1 The detection of T2 generation transgenic *Arabidopsis*

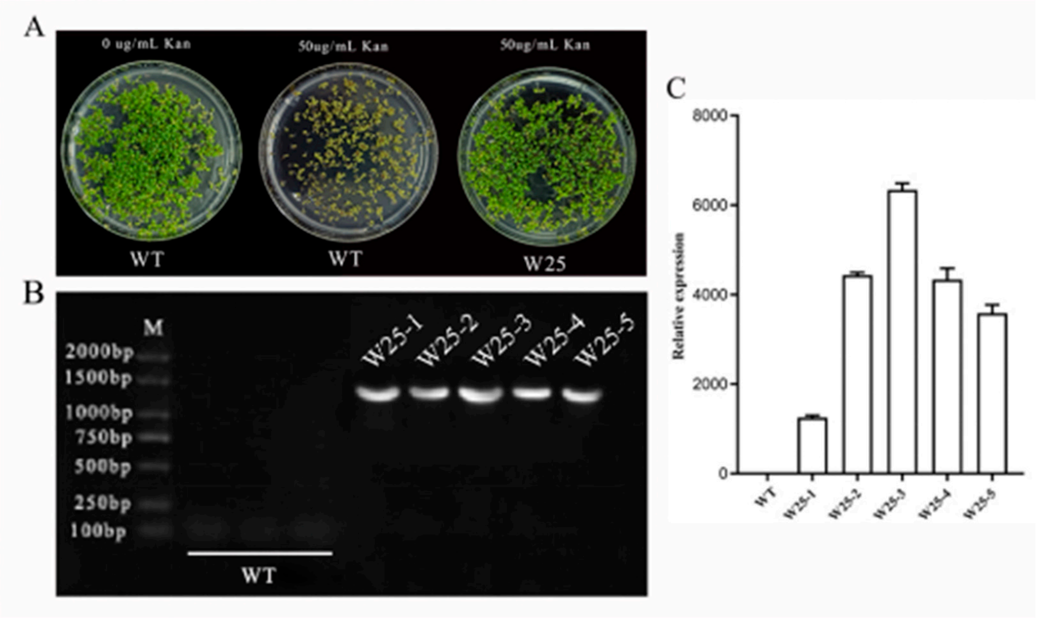

Figure S1 T2 generation transgenic *Arabidopsis* detection. (A) T2 generation resistance screening. (B) T2 generation transgenic *Arabidopsis* PCR detection; (C) qRT-PCR analysis of T2 transgenic *Arabidopsis* of W25. Data on the bars marked without the same lowercase letter indicated significant differences at $p \leqslant 0.05$.
