# Supplementary material for: Genome-Wide Identification of WRKY Gene Family and Functional Characterization of CcWRKY25 in Capsicum chinense
Source: Int J Mol Sci. 2023 Jul 13;24(14):11389. doi: 10.3390/ijms241411389 (PMC10379288; doi:10.3390/ijms241411389)
Supplement: Supplementary file 1 [file ijms-24-11389-s001.zip › Table S1 Primer sequences.pdf]

Table S1 Primer sequences

Primer	Sequence (5′-3′)	Tm/℃	Use
CcActin-QF	GTCCATCTGCTCTCTGTTG	58	qRT-PCR
CcActin-QR	CACCCCAAGCACAATAAGAC	58	qRT-PCR
CA06g13580-QF	TCCAAAACCTCTAAATAGTCCCCA	58	qRT-PCR
CA06g13580-QR	CCGACCACCATTACTATCACCC	58	qRT-PCR
CA07g10930-QF	CTGGAAGATGGTTACAGATGGAGA	58	qRT-PCR
CA07g10930-QR	TCGGCAAAGGGAAGGCAC	58	qRT-PCR
CcWRKY25-F	ACTCCAAACAAAACCAAGTCAAAAC	60.5	Clone
CcWRKY25-R	CGACTTGTGCTCCTTTCATTACC	60.5	Clone
*CcWRKY25*Q-F	ATTATCAAGAGCCCACAAAGCA	58	qRT-PCR
*CcWRKY25*Q-R	TACCTTTTACTTGTTTCTGCCCAT	58	qRT-PCR
pB: *CcWRKY25*-F	gagaacacgggggactctagaATGGCTGCTTCAAGTTTCTCAT TTC	60.5	Overexpression
pB: *CcWRKY25*-R	gagaacacgggggactctagaTCAGCAAAGCAATGACTCCAT AAAC	60.5	Overexpression
AtActin-QF	TCGCTGACCGTATGAGCAAAG	57	qRT-PCR
AtActin-QR	TGTGAACGATTCCTGGACCTG	57	qRT-PCR
AtPAL2-QF	AGACAAAAGTGGCGGTTACTACG	57	qRT-PCR
AtPAL2-QR	TAACGCTGCCTCCTACGGT	57	qRT-PCR
AtPAL4-QF	GAAATCACTCCGTGCCTCCC	57	qRT-PCR
AtPAL4-QR	CAGACATAACTTCCGATAAAACAGC	57	qRT-PCR
AtC4H-QF	TCTCAACCACCGTAATCTCGTC	57	qRT-PCR
AtC4H-QF	CCAATGCTCGCCGTAAACA	57	qRT-PCR
At4CL1-QF	GAACCCGAATCTTTATTTCCACAG	57	qRT-PCR
At4CL1-QR	GCCACCGTCACTTTACACCTC	57	qRT-PCR
At4CL2-QF	TGAAAGGCTATCTCAATGACCC	57	qRT-PCR
At4CL2-QR	TGGCGACGACAGCAACAT	57	qRT-PCR
At4CL3-QF	CCGTCGTCTCCGATGATGT	57	qRT-PCR
At4CL3-QR	TGTTTCGCCGTAGGTGTAGC	57	qRT-PCR
At4CL5-QF	TTGCCGTTGCTTACCTTGG	57	qRT-PCR
At4CL5-QR	CGTTGTCACCGTCATCGTCTAG	57	qRT-PCR
AtCHI-QF	CTCTTACGGTTGCGTTTTCG	57	qRT-PCR
AtCHI-QR	CGTCCTTGTTCTTCATCATTAGC	57	qRT-PCR
AtCHS-QF	CGTGTCCTCGTTGTCTGCTC	57	qRT-PCR
AtCHS-QR	CCTCAAATGTCCGTCTATGGC	57	qRT-PCR
AtF3H-QF	ATCATCGGAAACCTCCCTCA	57	qRT-PCR
AtF3H-QR	GCGAAATTGGCGTCGTGTAT	57	qRT-PCR
*AtFLS*1-QF	TCCCCGTGAAAAGATTGTTG	57	qRT-PCR
*AtFLS*1-QR	CTTGCGGTAACTGTAATCCTTGA	57	qRT-PCR
AtC3H-QF	GATGGACACGACAGCGATAACA	57	qRT-PCR
AtC3H-QR	GCAAGTAAGGTAAGCGGGAGAA	57	qRT-PCR
*AtCAD*1-QF	TAAAGGAGGCTACTCTAGTCACATTG	57	qRT-PCR
*AtCAD*1-QR	ACGCATCATAGGAGCATAAACC	57	qRT-PCR

Primer	Sequence (5′-3′)	Tm/℃	Use
*AtCAD*2-QF	GGCTTGGTGCTGATTCGTT	57	qRT-PCR
*AtCAD*2-QR	TCCAATCTGACTTCCTCCCAC	57	qRT-PCR
*AtCAD*3-QF	TCGGTGCTGATTCGTTTCTT	57	qRT-PCR
*AtCAD*3-QR	TCCAATCTGACTTCCTCCCAC	57	qRT-PCR
*AtCCR*1-QF	TCCATTGGTGCCGTCTACAT	57	qRT-PCR
*AtCCR*1-QF	CGTGGTAAAGACTGGCGTTG	57	qRT-PCR
AtCCOAOMT-QF	CAAATCAAGCTACGTCAACTTCC	57	qRT-PCR
AtCCOAOMT-QF	CGCCACAAAACCAAACCAC	57	qRT-PCR
*At*COMT-QF	GTTGGTGGTGGCATTGGTG	57	qRT-PCR
*At*COMT-QR	CCTCTGGAAGTGACTCGTAGCA	57	qRT-PCR
AtHCT-QF	CCACCGAGACACCAATCACTA	57	qRT-PCR
AtHCT-QR	GAACACCAGCACCGTTACAATC	57	qRT-PC
pT: *CcWRKY25*-F	gtgagtaaggttaccTTCTTTCACCGACCTTCTTGC	60	VIGS
pT: *CcWRKY25*-R	cgtgagctcggtaccCATCCTGCTTTGTGGGCTCT	60	VIGS
CcAT3-F	GCTTACAAGGACAGCGGCA	58	qRT-PCR
CcAT3-R	GAAGGAAGTTGAGGTGGCATACA	58	qRT-PCR
CcKAS-F	TCGGCTATTGGTGTTGGTGGT	58	qRT-PCR
CcKAS-R	CACCTCCCAAGTATTCCGCTA	58	qRT-PCR
CcBCAT-F	TTCAAGGAAGGAACAGCACCA	58	qRT-PCR
CcBCAT-R	TATCCGTTCGCCTTTGCTTT	58	qRT-PCR
CcPAMT-F	TGGATTTGGAAGACTTGGGACA	58	qRT-PCR
CcPAMT-R	GCTTACAAGGACAGCGGCA	58	qRT-PCR
CcPAL-F	TGTCCCGTTGTCCTACATTGCT	58	qRT-PCR
CcPAL-R	CTCGGGCTTTCCATTCATCAC	58	qRT-PCR
CcCA4H-F	TCGGTGTTGGTAGGAGGAGTTG	58	qRT-PCR
CcCA4H-R	TGCCCTGGAGGAGGAAACA	58	qRT-PCR
Cc4CL-F	CTTCTTCTCAACCATCCCAACA	58	qRT-PCR
Cc4CL-R	ACGAAATCCTTGACTTCATCCTC	58	qRT-PCR
CcCOMT-F	TAGCACATAACCCAGGAGGCA	58	qRT-PCR
CcCOMT-R	CACAGCACACCTTACGGAATCT	58	qRT-PCR
CcCS-QF	TTGGCTCGCGTATAATGACTT	58	qRT-PCR
CcCS-QR	TGCCGCTGGAATAACACCTC	58	qRT-PCR
Cc*CAD*-QF	TATGGCACCAGAACAAGCAG	58	qRT-PCR
Cc*CAD*-QR	CTCCAAGTCCCAATATTCCAC	58	qRT-PCR
Cc*CCR*1-QF	GATCCAGAACAAATGGTGGAGC	58	qRT-PCR
Cc*CCR*1-QR	CCAGCAAGTCTCGTCCACAAC	58	qRT-PCR
Cc*CCR*2-QF	AATGGTGGAGCCAGCAGTTAT	58	qRT-PCR
Cc*CCR*2-QR	GACAGCTCCAATTGAGGAAGTG	58	qRT-PCR
