# Supplementary material for: Genome-Wide Identification of WRKY Gene Family and Functional Characterization of CcWRKY25 in Capsicum chinense
Source: Int J Mol Sci. 2023 Jul 13;24(14):11389. doi: 10.3390/ijms241411389 (PMC10379288; doi:10.3390/ijms241411389)
Supplement: Supplementary file 1 [file ijms-24-11389-s001.zip › Table S2 Basic information on WRKY transcription factors of C.pdf]

Table S2 Basic information on *WRKY* transcription factors of *C. chinense*

Gene ID	Rename	Physical position		Amino acids /aa	Molecular weight /Da	pI	Subcellular localization
		Chromosome	Start-terminus sites				
BC332_20348_mrna	*CcWRKY1*	1	36308-51984	255	28804.77	9.35	Nucleus
BC332_07456_mrna	*CcWRKY2*	3	84774742-84783213	616	66538.80	6.23	Nucleus
BC332_05995_mrna	*CcWRKY3*	2	166378711-166381304	378	41986.85	8.22	Nucleus
BC332_21231_mrna	*CcWRKY4*	8	138776205-138777458	223	25052.45	6.22	Nucleus
BC332_27666_mrna	*CcWRKY5*	11	21574210-21579280	491	53750.29	6.81	Nucleus
BC332_26239_mrna	*CcWRKY6*	10	203654835-203657765	464	51333.71	9.47	Nucleus
BC332_25775_mrna	*CcWRKY7*	10	148383749-148385945	382	42773.57	6.46	Nucleus
BC332_31321_mrna	*CcWRKY8*	12	221395467-221396894	304	33432.36	9.12	Nucleus
BC332_00163_mrna	*CcWRKY9*	1	2551035-2562488	247	27907.08	5.91	Nucleus
BC332_05111_mrna	*CcWRKY10*	2	150829065-150831579	548	59550.85	6.90	Nucleus
BC332_18978_mrna	*CcWRKY11*	7	180224745-180228814	526	58352.32	6.22	Nucleus
BC332_03504_mrna	*CcWRKY12*	2	68944868-68946668	219	24264.82	9.58	Nucleus
BC332_31327_mrna	*CcWRKY13*	12	221520630-221522187	328	35905.08	9.11	Nucleus
BC332_00268_mrna	*CcWRKY14*	1	4084058-4085764	372	42306.88	5.85	Nucleus
BC332_31320_mrna	*CcWRKY15*	12	221376750-221381740	272	30645.98	9.21	Nucleus
BC332_02470_mrna	*CcWRKY16*	1	191946568-191948995	330	37026.51	6.08	Nucleus
BC332_23375_mrna	*CcWRKY17*	9	42067585-42069534	214	24121.03	8.26	Nucleus
BC332_20450_mrna	*CcWRKY18*	8	8055073-8056742	331	35757.94	9.80	Nucleus
BC332_34107_mrna	*CcWRKY19*	NA	54044-58678	418	46993.26	7.85	Nucleus
BC332_07394_mrna	*CcWRKY20*	3	62002983-62005547	300	34655.65	5.85	Nucleus
BC332_04245_mrna	*CcWRKY21*	2	131518275-131520003	321	36359.41	6.73	Nucleus
BC332_20350_mrna	*CcWRKY22*	8	3781832-3783574	364	41265.65	6.22	Nucleus
BC332_19791_mrna	*CcWRKY23*	7	225163160-225166675	747	81158.18	5.95	Nucleus
BC332_31325_mrna	*CcWRKY24*	12	221469539-221471028	303	33542.42	8.73	Nucleus
BC332_21563_mrna	*CcWRKY25*	8	173822025-173823936	330	37177.80	5.90	Nucleus
BC332_32647_mrna	*CcWRKY26*	NA	927922-930850	464	51543.90	9.58	Nucleus
BC332_31328_mrna	*CcWRKY27*	12	221526473-221528037	277	30605.43	9.53	Nucleus
BC332_19439_mrna	*CcWRKY28*	7	215024124-215038310	297	31411.75	6.18	Nucleus
BC332_18081_mrna	*CcWRKY30*	7	9240329-9248625	456	49741.78	9.05	Nucleus
BC332_20349_mrna	*CcWRKY31*	8	3773584-3777220	307	34558.20	7.66	Nucleus
BC332_28231_mrna	*CcWRKY32*	11	108141570-108143030	170	19605.29	9.50	Nucleus
BC332_06185_mrna	*CcWRKY33*	2	169294454-169295978	160	18575.88	9.45	Nucleus
BC332_33094_mrna	*CcWRKY34*	NA	403319-405163	339	37987.08	9.76	Nucleus
BC332_20351_mrna	*CcWRKY35*	8	3785892-3787500	281	32009.62	8.70	Nucleus
BC332_11915_mrna	*CcWRKY36*	4	225094831-225096315	351	38781.73	9.67	Nucleus
BC332_19890_mrna	*CcWRKY37*	7	226606299-226611823	583	63087.19	7.93	Nucleus
BC332_24379_mrna	*CcWRKY38*	9	245785900-245789969	606	66677.73	6.32	Nucleus
BC332_16509_mrna	*CcWRKY39*	6	217866888-217868385	361	39758.40	8.42	Nucleus
BC332_08895_mrna	*CcWRKY40*	3	246864717-246868512	550	61118.11	8.17	Nucleus
BC332_04408_mrna	*CcWRKY41*	2	135650434-135651876	306	34295.12	5.85	Nucleus

Gene ID	Rename	Physical position		Amino acids /aa	Molecular weight /Da	pI	Subcellular localization
		Chromosome	Start-terminus sites				
BC332_31593_mrna	*CcWRKY42*	NA	369481-372422	640	69603.83	6.43	Nucleus
BC332_23442_mrna	*CcWRKY43*	9	57488795-57492472	546	60478.28	8.10	Nucleus
BC332_08364_mrna	*CcWRKY44*	3	231673998-231674783	261	29419.12	6.31	Nucleus
BC332_32474_mrna	*CcWRKY45*	NA	256249-258355	498	55440.33	6.09	Nucleus
BC332_00165_mrna	*CcWRKY46*	1	2600409-2602960	218	25126.40	9.08	Nucleus
BC332_13551_mrna	*CcWRKY47*	5	155534269-155543016	230	25353.96	5.89	Nucleus
BC332_25469_mrna	*CcWRKY48*	10	72993366-73000272	315	36159.48	6.36	Nucleus
BC332_31324_mrna	*CcWRKY49*	12	221424066-221425345	255	27607.03	10.14	Nucleus
BC332_10771_mrna	*CcWRKY50*	4	64942506-64951288	487	54013.51	6.77	Nucleus
BC332_05559_mrna	*CcWRKY51*	2	159421746-159423199	324	35764.71	9.63	Nucleus
BC332_24089_mrna	*CcWRKY52*	9	214737683-214739678	330	36980.79	6.50	Nucleus
BC332_33123_mrna	*CcWRKY53*	NA	456232-464785	718	79413.31	8.16	Nucleus
BC332_24920_mrna	*CcWRKY54*	10	3807861-3810851	760	82646.36	6.82	Nucleus
BC332_14611_mrna	*CcWRKY55*	6	2758655-2760586	344	38628.63	9.59	Nucleus
BC332_26043_mrna	*CcWRKY56*	10	187385869-187388907	232	26729.07	7.62	Nucleus
BC332_03416_mrna	*CcWRKY57*	2	53330966-53335736	445	49221.38	5.87	Nucleus
BC332_14356_mrna	*CcWRKY58*	5	234303371-234306738	504	56581.09	7.17	Nucleus
BC332_08266_mrna	*CcWRKY59*	3	224295161-224296393	336	38605.17	5.67	Nucleus
BC332_27634_mrna	*CcWRKY60*	11	20227698-20229166	320	35419.11	9.07	Nucleus
BC332_27642_mrna	*CcWRKY61*	11	20574719-20580301	307	33760.09	5.64	Nucleus
BC332_19744_mrna	*CcWRKY62*	7	224124556-224127710	319	36653.20	6.65	Nucleus
BC332_08319_mrna	*CcWRKY63*	3	228474750-228476098	391	43793.14	6.15	Nucleus
BC332_11349_mrna	*CcWRKY64*	4	196837600-196839730	223	25792.06	6.40	Nucleus
BC332_30500_mrna	*CcWRKY65*	11	107943116-107948116	233	27084.36	8.90	Nucleus
BC332_03843_mrna	*CcWRKY66*	2	110989910-110995739	455	49881.67	6.99	Nucleus
BC332_30517_mrna	*CcWRKY67*	11	109678178-109693367	186	21479.71	7.09	Nucleus
BC332_21090_mrna	*CcWRKY68*	8	106992258-106993621	166	19027.04	6.08	Nucleus
BC332_03008_mrna	*CcWRKY69*	1	237239869-237244154	239	26746.32	8.16	Nucleus
BC332_02652_mrna	*CcWRKY70*	1	207114788-207116426	244	27048.20	5.82	Nucleus
BC332_14014_mrna	*CcWRKY71*	5	225255306-225257798	308	34785.58	5.52	Nucleus
BC332_21532_mrna	*CcWRKY72*	8	170588740-170590246	414	45715.72	6.76	Nucleus
BC332_21277_mrna	*CcWRKY73*	8	150643592-150646243	264	30158.91	6.37	Nucleus
